# Linking Self-Incompatibility, Dichogamy, and Flowering Synchrony in Two *Euphorbia* Species: Alternative Mechanisms for Avoiding Self-Fertilization?

**DOI:** 10.1371/journal.pone.0020668

**Published:** 2011-06-02

**Authors:** Eduardo Narbona, Pedro L. Ortiz, Montserrat Arista

**Affiliations:** 1 Departamento de Biología Molecular e Ingeniería Bioquímica, Universidad Pablo de Olavide, Sevilla, Spain; 2 Departamento de Biología Vegetal y Ecología, Universidad de Sevilla, Sevilla, Spain; Trinity College Dublin, Ireland

## Abstract

**Background:**

Plant species have several mechanisms to avoid selfing such as dichogamy or a self-incompatibility response. Dichogamy in a single flower may reduce autogamy but, to avoid geitonogamy, plants must show flowering synchronization among all their flowers (i.e. synchronous dichogamy). It is hypothesized that one species would not simultaneously show synchronous dichogamy and self-incompatibility because they are redundant mechanisms to reduce selfing; however, this has not been accurately assessed.

**Methodology/Principal Findings:**

This expectation was tested over two years in two natural populations of the closely related Mediterranean spurges *Euphorbia boetica* and *E. nicaeensis*, which completely avoid autogamy by protogyny at the cyathia level. Both spurges showed a high population synchrony (Z<79), and their inflorescences flower synchronously. In *E. nicaeensis*, there was no overlap among the cyathia in anthesis of successive inflorescence levels and the overlap between sexual phases of cyathia of the same inflorescence level was uncommon (4–16%). In contrast, *E. boetica* showed a high overlap among consecutive inflorescence levels (74–93%) and between sexual phases of cyathia of the same inflorescence level (48–80%). The flowering pattern of both spurges was consistent in the two populations and over the two successive years. A hand-pollination experiment demonstrated that *E. nicaeensis* was strictly self-compatible whereas *E. boetica* was partially self-incompatible.

**Conclusions/Significance:**

We propose that the complex pattern of synchronized protogyny in *E. nicaeensis* prevents geitonogamous crosses and, consequently, avoids selfing and inbreeding depression. In *E. boetica*, a high probability of geitonogamous crosses may occur but, alternatively, this plant escapes selfing through a self-incompatibility response. We posit that synchronous dichogamy and physiological self-incompatibility do not co-occur in the same species because each process is sufficiently effective in avoiding self-fertilization.

## Introduction

Angiosperms with hermaphroditic flowers have the capacity to self-fertilize. Although self-fertilization may be advantageous in some circumstances, in general, it has negative consequences for the individuals because they are more likely to express recessive or partially recessive deleterious mutations. Therefore, the fitness of the offspring produced by selfing can be lower than that of offspring produced by outcrossing (i.e. inbreeding depression) [1], [2]. Plant species have several mechanisms to avoid selfing and thus reduce inbreeding depression. These “anti-selfing mechanisms” can be classified into two main groups depending on whether they act before or after pollination [3], [4]. Pre-pollination mechanisms are based on floral display and design, and include spatial (i.e. herkogamy) or temporal (i.e. dichogamy) separation of male and female functions [5]. Another form of spatial separation is found in monoecious species in which pistillate and staminate unisexual flowers appear in the same individual [5], [6]. Post-pollination mechanisms are based on a self-incompatibility (SI) response in which self- and nonself-pollen are recognized by the plant that selectively rejects the self-pollen growth [2], [5], [7]. SI is genetically based (one or few S-loci) and it is proposed to be the most effective system for avoiding self-fertilization in flowering plants [7]. In spite of the high diversity of pre- and post-pollination mechanisms and their broad distribution among angiosperms, in a non-negligible number of cases, both mechanisms allow some degree of self-fertilization because they are imperfect or incomplete [6]–[11].

There exists considerable literature about the functional significance and the evolutionary implications of dichogamy [6,9,12,13, and references therein]. Depending on the criteria used, various types of dichogamy have been proposed [6], [9]: (1) protandry and protogyny can be defined according to which of the sexual phases are expressed earlier; (2) complete or incomplete if overlap between sexual phases exists; (3) intrafloral or interfloral in plants with unisexual flowers; and (4) synchronous if, pollen and stigma presentation of all flowers in anthesis are in synchrony, hemisynchronous if the synchrony is only among the flowers of the same inflorescence, and asynchronous if flowering is not synchronized. Two main selective forces can be proposed to explain the frequent occurrence of dichogamy among angiosperms [6], [9], [14]. First, dichogamy is thought to have evolved to reduce interference between pollen and stigma presentation within or among flowers (i.e. pollen discounting and ovule or seed discounting) [1], [6], [9]. Second, as explained above, dichogamy may simply be a mechanism to avoid self-fertilization [6]. To date, both hypotheses are widely accepted, but few experimental studies present data that try to support this [15]–[18]. By developing a population genetic model, Sargent et al. [14] found that both anther–stigma interference and selfing avoidance can lead to the evolution of dichogamy.

Plants with complete intrafloral dichogamy can totally avoid autogamy; moreover, if dichogamy is synchronous, geitonogamy is avoided as well [19], [20]. Some authors have hypothesized that if dichogamy can promote outcrossing and reduce self-pollination, species do not need other redundant mechanisms to avoid self-fertilization as SI systems [6], [21], [22]. However, this prediction has not been proven. For example, Lloyd and Webb [6] reported some plants with both dichogamy and SI, and Bertin [21] showed that dichogamy is equally common among self-compatible and self-incompatible angiosperms. Probably, however, dichogamy and SI are incomplete in these species (see above); thus, both characteristics may complement each other to reduce selfing [5], [6], [8]. In fact, species with synchronous and hemisynchronous dichogamy, which clearly favors xenogamy, are mostly self-compatible [23].

Thus, a fundamental aspect to determine if dichogamy and SI are two mutually exclusive mechanisms is to analyze the effectiveness of both mechanisms to avoid selfing. To test the effectiveness of dichogamy as an anti-selfing mechanism, we need to study the dichogamy of a flower and its synchronization with all the flowers on the plant [24]. To our knowledge, only three exhaustive studies have analyzed dichogamy and its synchrony with all the flowers of the plant [20], [25], [26], but the degree of SI of these species is not accurately known. In this paper, the pre- and post-pollination mechanisms to avoid selfing in two natural populations of *Euphorbia boetica* Boiss. as well as two natural populations of *E. nicaeensis* All. are investigated, both of whom are Mediterranean perennial spurges which are phylogenetically very close [27], and show complete intrafloral protogyny (referred within cyathia, see below) and interfloral dichogamy [28]–[30]. Thus, assuming that synchronous dichogamy and an SI system are redundant mechanisms [6], [22], we hypothesized that both spurges would present only one of these two efficient mechanisms. To address this question, we specifically consider the following objectives: (1) to assess the flowering phenology and duration, (2) to study the interfloral dichogamy at the inflorescence and plant levels, (3) to test if these flowering parameters are consistent among years and populations, and (4) to determine experimentally the presence of an SI system.

## Materials and Methods

### Study species

*Euphorbia boetica* Boiss. is endemic to the southern half of the Iberian Peninsula and grows in coastal pinewoods on sandy soils at altitudes of 0–100 m [31]. *Euphorbia nicaeensis* All. subsp. *nicaeensis* has a circum-Mediterranean distribution and prefers calcareous soils and grows in dry, sunny places at altitudes above 600 m in southern Spain [31]. The two species are not sympatric as they do not occur in the same stands.

**Figure 1:**
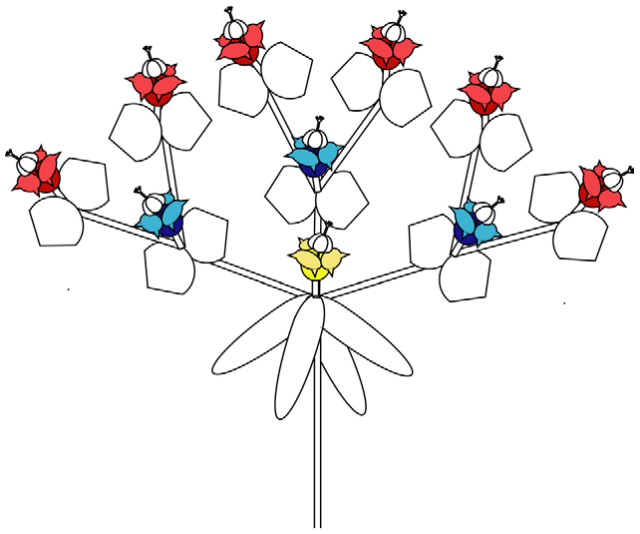
Diagrammatic representation of the inflorescences of *E. boetica* and *E. nicaeensis* showing the flowering order of the cyathia (levels), and the cyathia used for the intraindividual synchrony studies. Cyathia from different inflorescence levels are represented in different colors. First inflorescence level, yellow cyathium; second inflorescence level, blue cyathia; third inflorescence level, red cyathia. For simplification, we only show three inflorescence levels, but several inflorescence levels can be developed. Similarly, in the first branching (first inflorescence level) up to ten cyathia can spring from the terminal cyathium. In the following branching (second inflorescence level and higher) only two cyathia are bore from each terminal cyathium. In the study of flowering overlap among successive inflorescence levels, cyathia of different colors were compared. In the study of flowering overlap among cyathia of the same inflorescence level, cyathia of the same color were compared. Cyathia open acropetally.

*Euphorbia boetica* and *E. nicaeensis* are perennial herbaceous shrubs that branch at the base and produce numerous floral stems that bear a terminal inflorescence. Inflorescences present cyathia arranged in dichasia or pleiochasia (i.e. more than two cyathia spring from the terminal cyathium). The inflorescence is compound with several levels of branching, hereafter inflorescence levels, which bloom sequentially (Fig. 1). In the first branching, up to ten cyathia can develop (Inflorescence level 2 in Fig. 1), but in the following branching only two cyathia spring from each terminal cyathium (inflorescence level 3 in Fig. 1). The number of inflorescence levels that developed in *E. boetica* and *E. nicaeensis* are extremely variable and range between four and eight and three and five, respectively [29], [30]. Plants of *E. boetica* produce a mean of 70 cyathia per inflorescence and 722 per plant, whereas *E. nicaeensis* produce 62 and 382 cyathia, respectively [32]. The typical cyathia of *Euphorbia* can be functionally considered as a bisexual flower, although in fact they comprise a central pistillate flower surrounded by five groups of staminate flowers within a cup-like involucre [33], [34]. As the pistillate flower develops before the males, each cyathium is functionally a protogynous bisexual flower; thus the term intrafloral protogyny is used in this paper to refer to protogyny within cyathia [28]–[30]. Both species show no overlap between female and male phases within cyathia and the mean duration of the sexual phases is 4 and 12 days (female and male phases, respectively) for *E. boetica* and 3 and 11 days for *E. nicaeensis* [29]–[30]. The two spurges are functionally andromonoecious and produce male cyathia at the first levels of the inflorescence, and hermaphrodite cyathia at the remaining levels; however, *E. boetica* also produces some male cyathia at the last levels [29]–[30]. Cyathia of both species were actively visited by a taxonomically diverse array of insects (more than 100 taxa in each species) [32].

### Study sites

Two contrasting populations of *E. boetica* (Hinojos and El Gandul) and two of *E. nicaeensis* (La Camilla and Aracena) were studied in southern Spain. The Hinojos population (80 m asl; 37° 16′ N, 6° 25′ W) is within a mixed woodland of *Pinus pinea* L. and *Quercus suber* L. The Gandul population (40 m asl; 37° 20′ N, 5° 47′ W) is located on an abandoned farmland without tree cover. The La Camilla population (800 m asl; 36° 47′ N, 5° 24′ W) is in the Sierra de Grazalema Natural Park on sparse forest of *Quercus ilex* L. and *Ceratonia siliqua* L. The Aracena population (700 m asl; 37° 53′ N, 6° 33′ W) is situated on a cultivated woodland of *Castanea sativa* Mill.

### Flowering phenology

Flowering phenology was studied in three inflorescences from each of 54 plants of *E. boetica* (27 from each Hinojos and El Gandul populations) and from 51 plants of *E. nicaeensis* (28 and 23 from La Camilla and Aracena, respectively). The study was conducted in 1999 and 2000 during the whole flowering period. Unfortunately, in 1999, plants of the Aracena population were eaten by goats in the middle of the blooming period and data were not available. Plants were revisited every 7–10 days throughout the flowering period (8–9 censuses per year in Hinojos, 9–11 in El Gandul, 9 in La Camilla, and 7 in Aracena) to assess the number of blooming male and hermaphrodite cyathia.

### Inter- and intraindividual synchrony

Several indices to measure flowering synchrony within populations have been developed [35–36 and see references therein]. *Euphorbia boetica* and *E. nicaeensis* present a complex flowering phenology because cyathia show intra- and interfloral dichogamy and the inflorescences have several levels of branching. For these reasons, we employed the widely used Z index [37] to estimate the synchrony of the population, and other specific methods or indices to calculate the intraindividual synchrony. Augspurger's index measures the days in which an individual overlaps with the rest of the individuals of the population, and this is calculated through the following formula:
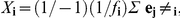
where **e_j_** is the number of days during which both individuals *i* and *j* flower synchronously (j ≠ i); ***f*** **_i_** is the number of days individual *i* is in flower, and **n** is the number of individuals in the population. ***X*** **_i_** may vary between 0 and 1; when ***X*** **_i_** = 0 no synchrony occurs, and when ***X*** **_i_** = 1 perfect synchrony occurs. The index of population synchrony (**Z**) is the average of the ***X*** **_i_** of all plants of the population.

To determine whether the inflorescences of a plant flower synchronously, we utilized a method modified from Thomson and Barrett [20]. We assessed the inflorescence level in which the cyathia are in anthesis and their state of development (bud, female phase, male phase, postmale phase) on three random inflorescences of 32–42 plants of *E. boetica* (Hinojos and El Gandul) and *E. nicaeensis* (La Camilla) along a linear transect. The censuses were carried out in 1999 (flowering peak in La Camilla) and in 2000 (beginning of flowering season and flowering peak-end flowering season in Hinojos; flowering peak in El Gandul; flowering peak in La Camilla).

The synchrony among successive inflorescence levels (i.e. flowering overlap) on the same inflorescence (Fig. 1) was estimated by recording the number of times in which two adjacent inflorescence levels are in anthesis simultaneously. This was measured in each tagged inflorescence on each census day during the total flowering period. Moreover, we estimated the percentage of inflorescences in which at least one overlap among successive inflorescence levels occurs in at least one census across the flowering period. If overlapping occurs, fertilization among cyathia of different inflorescence levels is possible because cyathia are in different sexual phases. With 0% overlap, no fertilization among cyathia of different inflorescence levels can occur. Based on observations of the body of pollinators [32], in this study we assumed that there is no carryover of viable pollen between cyathia flowering at different times.

In both *Euphorbia* species, generally all cyathia of the same inflorescence level bloom at the same time; however, synchrony among them may be not perfect. The asynchrony of flowering was estimated by considering the overlap among different sexual phases of the cyathia of the same inflorescence level (Fig. 1). We assessed the number of times that at least one flowering cyathium is at a different sexual phase (female or male) with respect to the rest of the cyathia. We estimated the percentage of inflorescences in which at least one overlap occurs in at least one census across the flowering period.

### Breeding system

The breeding system of both species was assessed experimentally in 1999 in the El Gandul population (*E. boetica*) and at La Camilla (*E. nicaeensis*). Prior to anthesis, inflorescences were bagged with polyester mesh bags (pore size ca. 2×2 mm) and tightened around the base of the peduncle. Only one inflorescence was used in each plant. In all experimental hand-pollinations, we used only cyathia that belonged to the inflorescence levels 5 and 3 in E. *boetica* and E. *nicaeensis*, respectively, because are the levels with the maximum cyathia production [30], [32]. One week later, when cyathia were in the female phase, the bags were removed and each inflorescence was randomly assigned to one of the following treatments. Xenogamy treatment was performed by applying fresh cross-pollen from two plants >10 m away to stigmas. Geitonogamy was tested by hand-pollinating stigmas with fresh pollen of another inflorescence of the same plant. Apomixis (except pseudogamy) was assessed through leaving the cyathia enclosed in bags during the anthesis. Spontaneous autogamy, i.e. automatic self-pollination, was not possible because cyathia of both species present complete protogyny [29], [30]. Additionally, a control treatment, i.e. open pollination, was left untreated. Flowers were bagged after pollination and allowed to senesce. We collected the mature fruits before dehiscence and viable seeds were counted in the laboratory. Viable seeds are easily distinguishable because the seed coat is brown whereas unviable seeds, i.e. seeds without embryo, are almost white and much lighter.

We calculated the self-compatibility index (SCI) for each species by dividing the number of fruits or seeds of the self-pollination (geitonogamy) treatment by those of the cross-pollination (xenogamy) treatment [38]. Lloyd and Schoen [8] considered species with an SCI lower than 0.75 as partially self-incompatible, and with an SCI higher than 0.75 as self-compatible.

To determine whether fruits or seeds produced by the geitonogamous crosses were due to an incomplete SI system or as the result of inbreeding depression in early stages [4], [39], the germination and pollen tube growth of pollen grains on the stigmata of both species were analyzed using the aniline blue staining method [4]. In each of the two years, six to eight cyathia were hand-pollinated by outcrossed or geitonogamuos pollen and were collected at 18, 24, and 48 h after pollination. Female flowers were fixed in formalin–alcohol–acetic acid for 24 h at 4°C, and then changed to 70% alcohol for storage. Later, plant material was soaked in 8 M NaOH for 5 min, washed with distilled water, stained with aniline blue (0.01%), and then inspected under an optical microscope at ×1600 and 2500 power with fluorescent light optics. With this method, callose produced on the pollen tube and on the stigma surface becomes fluorescent [40], [41].

### Statistical analysis

Differences in the duration of flowering and population synchrony between species and years were tested by means of Generalized Linear Models (GLM), assuming a log link function with a Poisson error distribution and a logit link function with a binomial error distribution [42], respectively; populations were nested within species. For these analyses, data from Aracena population in 1999 are treated as missing values.

To test for intraindividual synchrony between inflorescences of a plant, we utilized contingency tables to test the hypothesis that the bloom (inflorescence level and sexual phase) of an inflorescence is independent of those of other inflorescences of the plant. Contingency tables have two dimensions: phenological state of inflorescence *n* is compared to inflorescence *n+1* of the same plant. The levels within each dimension depends on the numbers of states of development of the cyathia (bud, female phase, male phase, postmale phase) in each population on the census day. In all contingency tables, some rows and columns were pooled to avoid bias of chi-square goodness of fit when at least one of the expected frequencies was less than five [43]–[44].

Differences between species in the percentage of overlap among different sexual phases of the cyathia of the same inflorescence level were analyzed by means of a binomial test to compare two proportions [42]. Data from different populations and years were analyzed separately. For this analysis, the sequential Bonferroni test was applied to control for experiment-wide type I error produced by multiple comparisons [45].

Fruit and seed set of experimental crosses were evaluated by means of GLMs assuming binomial error distribution and a probit link function. When the GLMs showed significant differences, the means of treatments were compared using *t* tests based on the standard errors calculated from the specific model.

For each response variable in the GLMs, we tested the link functions and error distributions that generated the smaller deviance in the model. Model selection was carried out using the Akaike's Information Criterion [42]. GLMs were carried out using SPSS version 18.0 (SPSS Inc., Chicago, IL, USA) with a hybrid of Fisher scoring and the Newton–Raphson algorithm. All other analyses were performed with R version 2.8.1 [46].

## Results

### Flowering phenology, duration and interindividual synchrony

**Figure 2:**
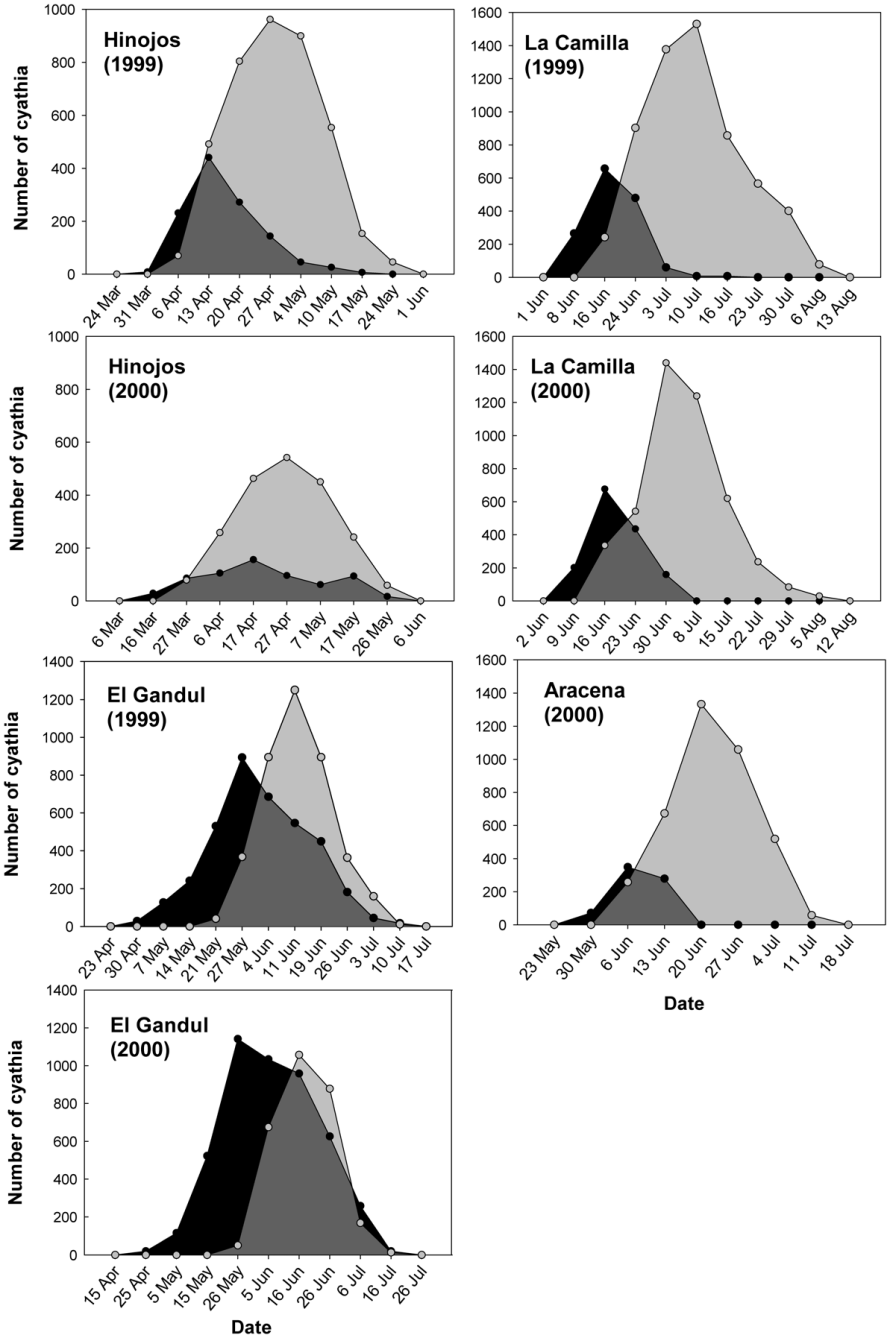
Flowering phenology of male (black circles) and hermaphrodites (gray circles) of E. boetica (left) and E. nicaeensis (right) in four populations over two years. Each point represents the mean of the population on the census date.

Flowering of *E. boetica* and *E. nicaeensis* took place from March to July in *E. boetica* and from May to August in *E. nicaeensis* (Fig. 2). In general, the hermaphrodite cyathia were much more numerous, with the exception of the El Gandul population of *E. boetica* (Fig. 2). For both species and in the two successive years, the flowering peak of male cyathia was reached 2–3 weeks earlier than those of hermaphrodite cyathia, with the exception of the Hinojos population in 1999 (Fig. 2).

The mean flowering duration of the individuals of the populations of *E. boetica* ranged between 44 (Hinojos population, 1999) and 66 days (El Gandul, 2000; Table 1). In *E. nicaeensis*, the mean flowering duration of the individuals ranged from 30 (Aracena population, 2000) to 42 days (La Camilla, 2000; Table 1). Thus, the flowering duration of *E. boetica* individuals was significantly longer than that of *E. nicaeensis* (Wald χ_1_^2^ = 15.7, *P*<0.0001); differences between years were not significant (Wald χ_1_^2^ = 0.0007, *P* = 0.93).

**Table 1:** Flowering duration and synchrony of *Euphorbia boetica* and *E. nicaeensis* plants during two years.

		Flowering duration (days)		Flowering synchrony	
Species/population	Year	Mean ± s.e.	Min.-Max.	Mean (Z) ± s.e.	Min.-Max. (X_i)_
*E. boetica*					
Hinojos	1999	44±1.9	21 – 56	0.90±0.012	0.77 – 0.98
Hinojos	2000	61±2.6	39 – 82	0.83±0.013	0.73 – 0.95
El Gandul	1999	58±2.0	43 – 79	0.90±0.015	0.73 – 0.99
El Gandul	2000	66±3.0	21 – 93	0.86±0.015	0.68 – 0.94
*E. nicaeensis*					
La Camilla	1999	42±1.9	21 – 58	0.80±0.015	0.66 – 0.95
La Camilla	2000	33±1.8	22 – 50	0.79±0.022	0.62 – 0.95
Aracena	2000	30±1.5	20 – 41	0.80±0.020	0.64 – 0.94

See materials and methods section for explanations of Z and X_i_.

In *E. boetica*, flowering phenology of each plant was continuous, i.e. there were some cyathia in anthesis during the entire flowering period of a plant. In contrast, in *E. nicaeensis*, flowering phenology of each plant was discontinuous and alternated between periods of flowering and no flowering.

Individuals of *E. boetica* and *E. nicaeensis* showed a high population synchrony (Z>0.83 and 0.79, respectively; Table 1). The population synchrony of *E. boetica* was not statistically different than that of *E. nicaeensis* (Wald χ_1_^2^ = 0.96, *P* = 0.62), but between years differences were significant (Wald χ_1_^2^ = 10.16, *P*<0.001).

### Intraindividual synchrony: among inflorescences

Results of contingency tests showed that the flowering phenology of an inflorescence was not independent of the rest of the analyzed inflorescences of a plant; thus, plants of both spurges presented synchronized flowering among inflorescences. In the Hinojos population (*E. boetica*), the inflorescences within each plant displayed the same phenological state both at the beginning of flowering (χ_9_^2^ = 17.78, n = 99, *P*<0.05) and at the end (χ_9_^2^ = 127.2, n = 126, *P*<0.0001). In El Gandul, the same results were observed in the only census that was carried out (χ_1_^2^ = 9.33, n = 96, *P*<0.01). In the La Camilla population (*E. nicaeensis*), the inflorescences within each plant displayed the same phenological state both in 1999 (χ_16_^2^ = 113.8, n = 63, *P*<0.0001) and in 2000 (χ_25_^2^ = 219.1, n = 96, *P*<0.0001).

### Intraindividual synchrony: flowering overlap among successive inflorescence levels

*Euphorbia boetica* showed a high degree of anthesis overlap among inflorescence levels in all the populations and years studied; however, neither of the analyzed inflorescences of *E. nicaeensis* showed anthesis overlap among different inflorescence levels (Table 2). In the Hinojos population of *E. boetica*, 93% and 74% of the inflorescences showed anthesis overlap among inflorescence levels in 1999 and 2000, respectively (Table 2); this overlapping mainly happened one to three times throughout the flowering period (Fig. S1). The frequency of overlapping decreased from the first levels to the last levels of the inflorescence (Fig. S2). In the El Gandul population, the majority of inflorescences also displayed overlapping between levels (89% in 1999 and 93% in 2000; Table 2). In most of these plants, the overlapping occurred from one to three times in both 1999 and 2000 (Fig. S1). Again, the frequency of overlapping decreased from the first to the last levels in both years but, in 2000, the overlapping in the last levels was not negligible (Fig. S2).

**Table 2:** Flowering overlap among inflorescence levels and among different sexual phases of cyathia of the same inflorescence level of *Euphorbia boetica* and *E. nicaeensis.*

			Overlap amonginflorescence levels		Overlap among cyathiaof the same levels	
Species/population	Year	Number of censuses	Inflorescences(%)	Censuses(%)	Inflorescences(%)	Censuses(%)
*E. boetica*						
Hinojos	1999	388	93	54	48	14
Hinojos	2000	362	74	15	67	18
El Gandul	1999	479	89	28	70	14
El Gandul	2000	471	93	25	80	24
*E. nicaeensis*						
La Camilla	1999	335	0	0	4	1
La Camilla	2000	352	0	0	7	2
Aracena	2000	206	0	0	16	5

See materials and methods section for more explanations.

### Intraindividual synchrony: flowering overlap among cyathia of the same inflorescence level

In *E. boetica* inflorescences, the overlapping among different sexual phases of the cyathia of the same inflorescence level was between 48% (Hinojos population, 1999) and 80% (El Gandul, 2000; Table 2). In contrast, in *E. nicaeensis* inflorescences, the overlapping between different sexual phases of cyathia was a rare event, ranging from 4% (La Camilla, 1999) to 16% (Aracena, 2000; Table 2). The overlapping between different sexual phases of the populations of *E. boetica* plants was statistically higher than those of the populations of *E. nicaeensis* in 1999 and 2000 (all binomial tests was statistically significant after Bonferroni correction, α = 0.05/6 = 0.0083). The same was true considering the number of censuses in which overlapping between different sexual phases occurred (all binomial tests was statistically significant after Bonferroni correction, α = 0.05/6 = 0.0083, for 1999 and 2000).

### Breeding system

None of the unpollinated bagged cyathia of both *E. boetica* and *E. nicaeensis* bore fruit; thus, apomixis (except pseudogamy) was discounted (Table 3). In *E. boetica*, the fruit set of the other three treatments was statistically different (Wald χ_2_^2^ = 26.52, *P*<0.0001; Table 3). Only 10.7% of the cyathia of the self-pollination treatment set fruits. In fact, only six of the 19 plants of this treatment developed any fruit. Self-pollination produced a significantly lower proportion of fruits than cross-pollination (Table 3), giving an SCI for fruit set of 0.13. Less fruit was set in the open-pollination treatment than in the cross-pollination treatment (Table 3). The seed sets of *E. boetica* were significantly different between treatments (Wald χ_2_^2^ = 14.40, *P*<0.001), and again the seed set of selfed cyathia was lower than those of the other two treatments (Table 3). Thus, the SCI for seed set was 0.55.

**Table 3:** Results of the breeding experiments on *E. boetica* and *E. nicaeensis*.

	Fruit set						Seed set					
	*E. boetica*			*E. nicaeensis*			*E. boetica*			*E. nicaeensis*		
Treatment	n	N	Mean ± s.e.	n	N	Mean ± s.e.	n	N	Mean ± s.e.	n	N	Mean ± s.e.
Open	260	20	61.6±5.3 a	256	15	78.2±5.4 a	161	17	73.5±3.0 a	156	15	63.5±4.9 a
Cross	45	15	82.8±6.1b	83	12	74.1±6.1 a	24	9	70.5±4.1 a	62	12	63.7±5.5 a
Self	67	19	10.7±5.4 c	63	10	66.8±6.7 a	8	6	38.9±5.0 b	38	10	60.1±6.0 a
Apomixis	78	15	0±0.0	94	12	0±0.0	-	-	-	-	-	-

n =  number of cyathia or fruits, N =  number of plants. Different letters indicate significant differences between means of treatments.

In *E. nicaeensis*, fruit sets from self, cross, and open-pollination treatments were not significantly different (Wald χ_2_^2^ = 1.56, *P* = 0.46; Table 3), and in this case the SCI for fruit set was 0.90. Seed sets were also similar between the three treatments (Wald χ_2_^2^ = 1.84, *P* = 0.40; Table 3), and the SCI for seed set was 0.94.

In both *Euphorbia* species, and as much in the selfed as in the crossed treatments, we observed that, in the cyathia collected at 18, 24, and 48 h after hand-pollination, the pollen germinated and the pollen tubes penetrated the stigmatic tissue (Fig. S3). In the self-pollinated stigmas of *E. boetica*, some nongerminated pollen grains were observed. Furthermore, in the germinated grains, brightly fluorescent regions on the surface of the stigma around the pollen tube were distinguished (Fig. S3A); this corresponds to callose deposits. We could not observe any pollen tubes penetrating the ovules in any of the treatments and species.

## Discussion

*Euphorbia boetica* and *E. nicaeensis* showed a very high population synchrony in relation to other species [35], [37], and this was consistent across years as well as populations. This high synchrony means that most of the blooming period of an individual coincides with those of the other plants of the population. The plants of *E. boetica* bloom continuously for several weeks, while the plants of *E. nicaeensis* display a discontinuous blooming, with periods of flowering alternating with non-flowering periods. This different flowering pattern occurs because there is a frequent overlap among cyathia in anthesis of successive inflorescence levels in *E. boetica*, whereas there is no overlap in *E. nicaeensis* and the anthesis of two successive levels is preceded by several days of nonflowering. Thus, on no single day of the *E. nicaeensis* flowering period, are all the plants of a population in flower.

In both spurges, male cyathia were more numerous than hermaphrodites at the beginning of the blooming period. This pattern was due both to the exclusive presence of male cyathia in the first levels of the inflorescence [29], [30] and the high population synchrony. In the El Gandul population of *E. boetica* and in both populations of *E. nicaeensis*, this situation was very noticeable, and for several days (up to three weeks in El Gandul) there were only male cyathia with no ovaries to fertilize in the entire population (Fig. 2). This rare flowering pattern has also been found in *Aralia hispida* and in *Datisca glomerata* (andromonoecious and androdioecious species, respectively) [20], [47]. The nonoverlap among male and female gametes at the beginning of the blooming period generates a temporal separation of staminate and pistillate functions not only at the individual level (called temporal dioecism; [23], [48]) but also at the population level. This apparent waste of resources would ensure the existence of a huge amount of available pollen in the population when the first ovules of hermaphrodite cyathia began anthesis [49].

Our results demonstrate that *E. boetica* and *E. nicaeensis* rely entirely on pollinators for reproduction because bagged cyathia did not produce fruits. Both spurges can produce fruits and seeds after geitonogamous crosses, but based on fruit and seed SCI, *E. nicaeensis* is a strictly self-compatible species, whereas *E. boetica* is a partially self-incompatible species [8]. The facts that in *E. boetica* most plants in the population did not develop fruits after selfing (68%), and that callose deposit were found around the sites where selfed pollen tubes penetrated the stigma, support the idea of the presence of an incomplete SI system [4], [40], [41], [50], [51]. Callose deposits and a decrease in the fruit and seed set of selfed crosses have been also found in *Euphorbia esula* [52]. On the other hand, in *E. boetica* (El Gandul), the cross-pollination treatment produced ca. 20% more fruit set than the open pollination treatment, suggesting that reproduction is pollen limited in this population [53].

In *E. nicaeensis*, there was no overlap among cyathia in anthesis of successive inflorescence levels and moreover, any overlap between sexual phases of cyathia of the same inflorescence level was markedly rare. This flowering pattern can be considered as a form of synchronous protogyny, as most of the inflorescences of a plant are also synchronized. Under these circumstances, the probability of natural geitonogamous fertilization in *E. nicaeensis* is extremely rare [16]. Although synchrony has been found in several species belonging to different families (e.g. Alstroemeriaceae and Rubiaceae; [16], [25]), the same complex flowering system of *E. nicaeensis* has only been found in species of the Araliaceae and Umbelliferae [6], [24], [48], [54], [55]. We therefore suggest that interfloral protogyny and the complex pattern of synchronized flowering in *E. nicaeensis* are effective mechanisms to reduce geitonogamous crosses and, consequently, to avoid selfing and inbreeding depression.

In contrast, *E. boetica* showed overlap among two or more inflorescence levels. Although the frequency of overlapping decreased from the first to the last levels, it was relatively high in the intermediate levels, which bore the highest number of cyathia [29]. In addition, overlap between sexual phases of cyathia of the same inflorescence level was common. Most insect pollinators of *E. boetica* typically visit all the cyathia in anthesis of an inflorescence [32], as is found in others species with umbellate inflorescences [26], including some spurges [56], [57]. The overlap of flowering, the pollinator behavior, and the great production of flowers [32] suggest that geitonogamous pollinations frequently occur in natural populations of *E. boetica* [58], [59]. However, in *E. boetica*, geitonogamous fertilizations are highly limited due to its partial-SI system.

It is noteworthy that synchronous dichogamy and SI were not simultaneously found in both *E. boetica* and *E. nicaeensis*. Barrett [24] proposed that, to exclude selfing, a synchronous dichogamy may have evolved in species of Araliaceae and Umbelliferae as an alternative to an SI mechanism, which is absent in these families. Using phylogenetic comparative methods, Routley et al. [22] showed that, at least at the family level, dichogamy and SI can evolve rapidly, and Loo et al. [60] have proposed that dichogamy may influence the high diversification in a genus of Arecaceae. We posit that synchronous dichogamy and physiological SI may have evolved independently in *Euphorbia* as two different ways to avoid selfing [16].

Euphorbiaceae, and specifically *Euphorbia*, has been mainly considered a self-compatible group [39], [61], [62]. However, several species show a high reduction of fruit set after geitonogamous crosses and they have been considered as a self-incompatible or partially self-incompatible species [52], [61], [63]. As the *Euphorbia* species with reported SI belong to different subgenera and sections [31], [64], it is plausible to think that SI or partial SI could have evolved independently at several times, as has been proposed in other families [65], [66]. Similarly, the SI found in *E. boetica* may have evolved to avoid inbreeding depression, which is not excluded by their pre-pollination anti-selfing mechanism. On the other hand, synchronous dichogamy may have originated in *E. nicaeensis* as a modification of the floral and flowering characteristics shared by all species of the *Euphorbia* subgenus *Esula*: intrafloral protogyny and cyathia arranged in compound pleiochasial inflorescences [28], [31]. However, given that only two species have been studied, our result should be considered with caution. Further studies in *Euphorbia*, specifically in section *Paralias*, which include the reconstruction of the evolutionary history of synchronous dichogamy and SI, and their phylogenetic associations [67], are required to elucidate if both characters are inversely associated and if they play a key role in the diversification of the group.

## Supporting Information

Figure S1 **Frequency histograms showing the number of times that two inflorescence levels in anthesis of the same inflorescence overlapped in two populations over two years in** ***E. boetica*** **.** Total number of censuses is shown in Table 2.
(TIF)

Figure S2 **Frequency histograms showing in which inflorescence levels the overlapping between two inflorescence levels occurs in two populations over two years in E. boetica.** Total number of censuses is shown in Table 2.
(TIF)

Figure S3 **Pollen germination in stigmas of** ***E. boetica*** **and** ***E. nicaeensis*** **.** Styles were fixed 24 h after pollination and stained with aniline blue. A, pollen germination after geitonogamous crosses in *E. boetica* (×2500). B, pollen germination after xenogamous crosses in *E. boetica* (×2500). C, pollen germination after geitonogamous crosses in *E. nicaeensis* (×1600). D, pollen germination after xenogamous crosses in *E. nicaeensis* (×1600). Bar  = 5 µm. Yellow arrows show fluorescent accumulations of callose on stigma cells around the pollen tube penetration.
(TIF)
