# Supplementary figures and images for: Linking Self-Incompatibility, Dichogamy, and Flowering Synchrony in Two Euphorbia Species: Alternative Mechanisms for Avoiding Self-Fertilization?
Source: PLoS One. 2011 Jun 2;6(6):e20668. doi: 10.1371/journal.pone.0020668 (PMC3107240; doi:10.1371/journal.pone.0020668)

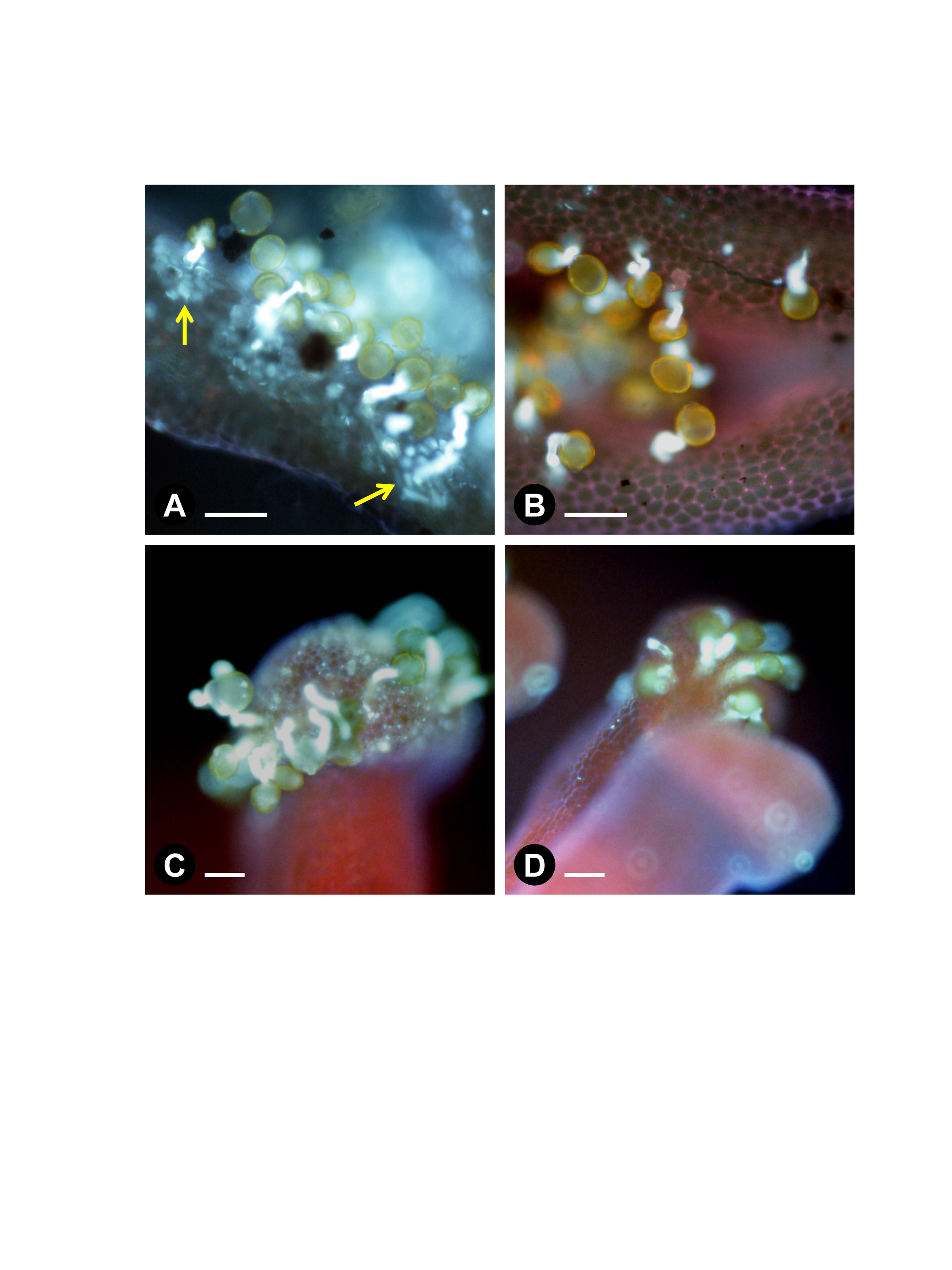

Supplement: Figure S3 — Pollen germination in stigmas of E. boetica and E. nicaeensis . Styles were fixed 24 h after pollination and stained with aniline blue. A, pollen germination after geitonogamous crosses in E. boetica (×2500). B, pollen germination after xenogamous crosses in E. boetica (×2500). C, pollen germination after geitonogamous crosses in E. nicaeensis (×1600). D, pollen germination after xenogamous crosses in E. nicaeensis (×1600). Bar = 5 µm. Yellow arrows show fluorescent accumulations of callose on stigma cells around the pollen tube penetration. (TIF) [file pone.0020668.s003.tif]
